# Bi–O–S Catalyst for Enhanced CO_2_ Electroreduction to Formic Acid: Catalyst–Substrate Engineering and Multi-Objective Optimization

**DOI:** 10.3390/molecules31203718

**Published:** 2026-10-16

**Authors:** Mithra Geetha, Zeyad M. Ghazi, Muhammad Arsalan, Muftah H. El-Naas

**Affiliations:** 1 Gas Processing Centre, College of Engineering, Qatar University, Doha P.O. Box 2713, Qatar; 2 Central Laboratories Unit, Qatar University, Doha P.O. Box 2713, Qatar

**Keywords:** formic acid, Bi–O–S catalyst, acid-treated Sn, bismuth electrocatalyst, Faradaic efficiency

## Abstract

Electrochemical CO_2_ reduction to formic acid is a promising approach for carbon valorization, but achieving both high productivity and Faradaic efficiency remains a major challenge. In this study, a Bi–O–S electrocatalyst was synthesized and deposited onto an acid-treated Sn substrate to fabricate a Bi–O–S/acid-treated Sn electrode. Structural, chemical, morphological, and textural characterization confirmed successful formation and integration of the Bi–O–S catalytic phase with the Sn surface. Acid treatment of the electrode surface enhanced the formic acid concentration from 170 mg L^−1^ for untreated Sn to 750 mg L^−1^. Central composite design coupled with response surface methodology was subsequently employed to optimize applied current density, catholyte concentration, and catalyst loading. Multi-objective optimization experimentally achieved 1020 mg L^−1^ formic acid and 67% Faradaic efficiency, compared with predicted values of 950 mg L^−1^ and 61%, respectively, corresponding to a production rate of 41 mg h^−1^. The electrode maintained stable performance for 33 h, demonstrating the potential of Bi–O–S/acid-treated Sn electrode engineering for efficient and optimized CO_2_-to-formic-acid conversion.

## 1. Introduction

The increasing atmospheric concentration of carbon dioxide (CO_2_), together with the need to reduce reliance on fossil-derived chemical manufacturing, has intensified research into technologies that can simultaneously mitigate CO_2_ emissions and generate value-added chemicals. Electrochemical CO_2_ reduction (CO_2_RR), particularly when powered by renewable electricity, offers an attractive carbon-utilization pathway by coupling CO_2_ conversion with storage of electrical energy in chemical bonds. Among the products accessible through CO_2_RR, formic acid/formate is particularly appealing because of its established applications in the chemical, pharmaceutical, agricultural, and textile sectors and its potential as a liquid hydrogen carrier [1,2,3,4].

CO_2_-to-formate/formic-acid conversion generally proceeds through a two-electron reduction pathway, although the dominant product and reaction environment depend strongly on electrolyte composition and pH. Achieving efficient CO_2_RR therefore requires selective activation of CO_2_ while suppressing the competing hydrogen evolution reaction and other reduction pathways. Catalyst composition and surface structure, together with electrode architecture, electrolyte environment, and operating conditions, collectively determine reaction kinetics, selectivity, and energy utilization. Accordingly, the development of practical CO_2_RR systems increasingly requires an integrated assessment of catalyst, electrode, and process parameters rather than optimization of Faradaic efficiency (FE) alone [3,4,5].

Bismuth (Bi)-based materials are attractive electrocatalysts for CO_2_-to-formate conversion because of their favorable two-electron selectivity and relatively low hydrogen-evolution activity [6,7,8]. Their performance depends strongly on oxidation state, crystal structure, particle size, defects, morphology, and local coordination. Accordingly, nano-structuring, defect engineering, phase modulation, heteroatom incorporation, and heterostructure formation have been widely explored to tailor Bi-based catalysts [7,8,9,10]. Formate formation is commonly associated with stabilization of oxygen-bound intermediates such as OCHO, making the Bi electronic and coordination environment important for CO_2_ activation, intermediate adsorption, and proton-coupled electron transfer [6,9,10].

Recent Bi-based CO_2_RR systems include metallic Bi, Bi oxides, bismuth oxy-compounds, heterostructures, and heteroatom-modified materials [8,9,10,11]. Sulfur incorporation is particularly relevant because it can modify the electronic structure, coordination environment, and surface properties of Bi [10]. However, ex situ characterization may not fully represent the active catalyst under CO_2_RR conditions because electrochemical reconstruction can alter the surface structure and chemical state. Therefore, linking Bi catalyst properties with electrode architecture and catalyst–substrate interactions is essential for developing practically effective CO_2_RR electrodes.

Beyond catalyst composition, the substrate and catalyst–substrate interface can strongly affect CO_2_RR by influencing electrical connectivity, catalyst adhesion, accessible surface area, charge transfer, and the local reaction environment [4,12]. Sn is particularly attractive for formate production because of its favorable CO_2_-to-formate selectivity [13,14,15,16]. Surface engineering of Sn can further modify its chemistry and morphology, providing a suitable platform for catalyst immobilization and interfacial charge transfer. Bi–Sn interfaces may additionally influence CO_2_ activation and intermediate adsorption [10,17]. However, the role of deliberate Sn surface modification in controlling immobilized Bi-based catalyst performance remains insufficiently established.

Achieving high formic acid productivity together with high FE remains another major challenge. Although increasing current density can enhance product formation, excessive current may promote hydrogen evolution and mass-transfer limitations. Catholyte concentration and catalyst loading can likewise affect CO_2_ availability, active-site accessibility, and competing reactions. Thus, optimization of these parameters should consider their coupled effects, since maximizing FE alone does not necessarily maximize formic-acid productivity [3,5,18].

Response surface methodology (RSM) provides an effective statistical framework for resolving such interactions and identifying operating conditions that simultaneously satisfy multiple performance objectives. This approach is particularly relevant to CO_2_RR, where catalyst properties, electrode architecture, mass transport, and electrochemical operating conditions are intrinsically coupled. Despite considerable advances in Bi-based CO_2_RR, the systematic integration of catalyst development with substrate engineering and multi-response process optimization remains comparatively limited. Moreover, assessments based predominantly on FE can overlook productivity and operational durability, which are critical for practical electrosynthesis [4,8,12].

In this work, a Bi–O–S catalyst was integrated with an acid-treated Sn substrate to construct a Bi–O–S/acid-treated Sn electrode for electrochemical CO_2_ conversion to formic acid. The effects of substrate modification, catalyst loading, applied current density, and catholyte concentration were systematically investigated and optimized using response surface methodology (RSM), with formic acid concentration and FE considered as complementary responses. Multi-objective optimization was used to identify experimental conditions that balance productivity and selectivity, followed by experimental validation and extended electrolysis to evaluate operational stability. The novelty of this study lies in integrating Bi–O–S catalyst engineering, acid-induced Sn surface modification, catalyst–substrate interfacial design, and multi-objective process optimization within a unified CO_2_-to-formic-acid platform. This integrated strategy provides a practical approach for simultaneously addressing productivity, selectivity, and operational stability in Bi-based CO_2_RR electrodes.

## 2. Results and Discussion

### 2.1. Structural and Physicochemical Characteristics of the Bi–O–S Catalyst

#### 2.1.1. Crystal Structure and Phase Composition

**Figure 1:**
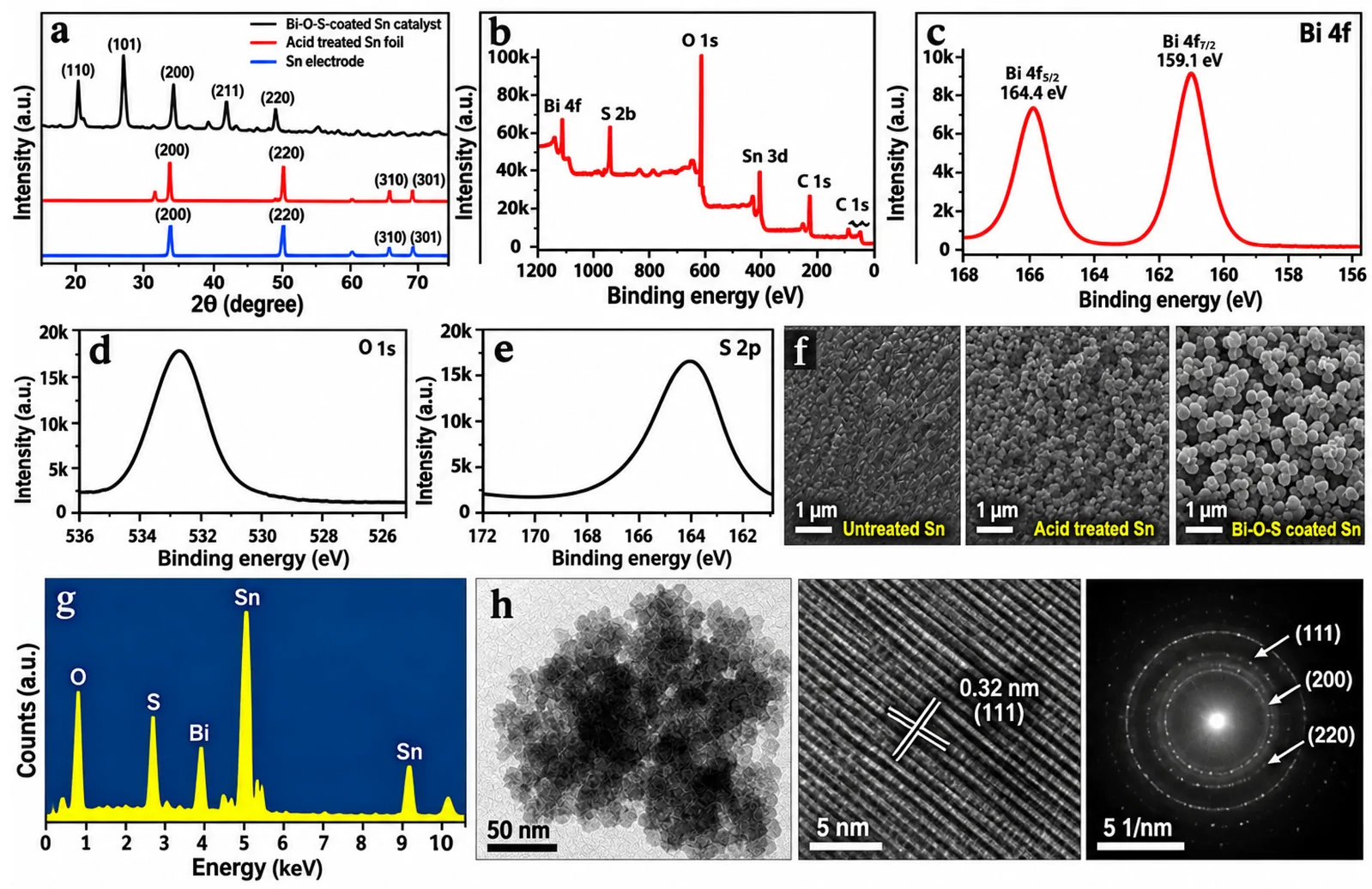
Structural, chemical, morphological, and compositional characterization of the Bi–O–S/AtSn electrode: (**a**) XRD patterns; (**b**) XPS survey spectrum; (**c**–**e**) high-resolution Bi 4f, O 1s, and S 2p spectra, respectively; (**f**) FESEM images of untreated Sn, acid-treated Sn, and Bi–O–S-coated Sn electrodes; (**g**) EDX spectrum showing the elemental composition of the Bi–O–S/AtSn electrode; and (**h**) TEM characterization of the Bi–O–S catalyst, including a TEM image, HRTEM image showing lattice fringes with an interplanar spacing of 0.32 nm corresponding to the (111) plane, and the corresponding SAED pattern confirming its crystalline nature.

The crystalline structure and phase composition of untreated Sn, acid-treated Sn (AtSn), and Bi–O–S/AtSn electrodes were examined by XRD (Figure 1a). Untreated Sn exhibited intense reflections at 2θ ≈ 31.92°, 44.66°, 44.92°, 55.29°, 62.48°, and 64.58°, characteristic of crystalline β-Sn. After sulfuric acid treatment, the Sn reflections decreased in intensity, while additional peaks at ≈31.56° and 42.86° appeared, indicating modification of the near-surface region and formation of oxygen- and sulfur-containing surface species. Following Bi–O–S deposition, new reflections at ≈18.87°, 21.68°, 24.41°, 27.79°, and 32.82° were observed and assigned to Bi-containing oxide and sulfate phases, including Bi_2_O_2_SO_4_, δ-Bi_2_O_3_, γ-Bi_2_O_3_, and Bi_2_(SO_4_)_3_. The identification of Bi_2_O_2_SO_4_ is consistent with its characteristic Bi–O and sulfate structural environments [19]. The coexistence of oxide- and sulfate-associated reflections supports a multiphase Bi–O–S-related crystalline structure.

The Bi–O-containing phases may influence CO_2_ adsorption and stabilization of reaction intermediates through their local Bi–O coordination environments. Bi oxide structures have been reported to promote selective CO_2_ conversion to formate, while catalyst morphology and exposed surface features can further affect electrochemically accessible sites and selectivity [20,21]. Importantly, Bi-based catalysts may undergo electrochemical reconstruction under CO_2_ reduction conditions, involving changes in Bi oxidation state, local coordination, and surface structure that can modify the active catalytic interface [22,23,24].

#### 2.1.2. Surface Chemical States and Bi Oxidation State

The surface elemental composition and chemical states of the Bi–O–S catalyst were examined by X-ray photoelectron spectroscopy (XPS), with the corresponding spectra presented in Figure 1b–e. The survey spectrum (Figure 1b) confirms the presence of Bi, O, and S as the major surface elements.

The high-resolution Bi 4f spectrum (Figure 1c) shows peaks at approximately 157.8 and 163.1 eV, assigned to Bi 4f_7_/_2_ and Bi 4f_5_/_2_, respectively, indicating predominantly oxidized Bi^3+^ and no evident metallic Bi^0^ contribution. The O 1s spectrum (Figure 1d) displays a dominant peak at approximately 529.8 eV, consistent with lattice oxygen in Bi–O-containing environments, while the S 2p spectrum (Figure 1e) shows a characteristic contribution at approximately 162.6 eV, confirming sulfur-containing species. These results support the Bi–O–S surface composition and are consistent with the oxide- and sulfate-containing phases identified by XRD.

The predominantly oxidized Bi state is relevant to CO_2_RR because Bi-based catalysts can undergo surface reconstruction under cathodic conditions, with residual Bi–O or positively polarized Bi environments potentially influencing OCHO-type intermediate formation and stabilization [22,23,24]. Accordingly, the XPS results represent the initial surface chemical state of the as-prepared catalyst and should not be interpreted as the definitive oxidation state of the catalytically active surface during prolonged electrolysis.

#### 2.1.3. Surface Morphology and Elemental Distribution

The surface morphology of the untreated Sn, acid-treated Sn (AtSn), and Bi–O–S/AtSn electrodes were examined by FESEM, with the corresponding micrographs and he EDX spectrum shown in Figure 1f and Figure 1g, respectively. The untreated Sn surface is relatively smooth and compact, with visible polishing marks. Acid treatment produces a considerably rougher and more irregular surface containing grooves and microscale depressions, indicating modification of the near-surface structure and providing additional sites for catalyst anchoring and increased interfacial area. After Bi–O–S deposition, the surface is covered by a densely distributed, flake-like and aggregated structure with interconnected voids. Higher-magnification images reveal closely packed irregular particles and flakes, which may increase accessible surface regions and influence interfacial mass transport. Such structured Bi-based surfaces, particularly those containing exposed edges and corners, have been associated with favorable CO_2_-to-formate electroreduction [21].

EDS analysis confirms successful catalyst deposition, with the Bi–O–S/AtSn electrode containing 62.42 wt% Bi, 27.74 wt% O, and 9.84 wt% S. In contrast, acid-treated Sn contains 6.73 wt% O and 0.67 wt% S, whereas untreated Sn is predominantly composed of Sn. The elemental maps show a broadly uniform distribution of Bi, O, and S across the catalyst layer, with no pronounced large-scale segregation, supporting relatively homogeneous Bi–O–S deposition and formation of a consistent catalyst–electrolyte interface.

#### 2.1.4. Nanostructure and Textural Properties

The nanoscale morphology and crystallinity of the synthesized Bi–O–S catalyst were further investigated by transmission electron microscopy (TEM), high-resolution TEM (HRTEM), and selected-area electron diffraction (SAED), as shown in Figure 1h. TEM image reveals aggregated nanoscale particles forming compact, interconnected clusters, indicating the formation of a hierarchical nanoparticulate structure. Although some degree of particle aggregation is evident, the individual nanoscale structural units remain distinguishable, confirming the successful formation of the Bi–O–S nanostructure. The HRTEM image provides direct evidence of the crystalline nature of the catalyst, with clearly resolved lattice fringes. An interplanar spacing of approximately 0.32 nm is observed and can be assigned to the (111) crystallographic plane of the Bi–O–S catalyst, in good agreement with the crystalline structure identified from the XRD analysis in Figure 1a. The corresponding SAED pattern exhibits distinct concentric diffraction rings, indexed to the (111), (200), and (220) planes, further confirming the polycrystalline nature of the synthesized catalyst. The agreement between the HRTEM lattice spacing, SAED diffraction rings, and XRD reflections provides complementary evidence for the crystalline Bi–O–S phase.

The textural properties of the synthesized catalyst were determined from N_2_ adsorption–desorption analysis using the BET method. The catalyst exhibited a specific surface area of 3.921 m^2^ g^−1^ and a total pore volume of 0.001476 cm^3^ g^−1^, as summarized in Table 1. The relatively low pore volume indicates that the material does not possess a highly developed porous network. Instead, the observed voids are more likely associated with spaces between aggregated particles and structural features within the catalyst layer.

### 2.2. Effect of Sn Surface Engineering and Bi–O–S Catalyst Deposition on CO_2_ Reduction

To distinguish the contributions of the Sn substrate treatment and Bi–O–S catalyst incorporation, the electrochemical performance of four electrode configurations was compared (Table 2) under identical operating conditions. The experiments were conducted using a 0.60 M catholyte at an applied current of 50 mA, with untreated Sn, acid-treated Sn (AtSn), Bi–O–S-coated untreated Sn, and Bi–O–S-coated acid-treated Sn evaluated as working electrodes.

#### 2.2.1. Comparison of Untreated and Acid-Treated Sn Electrodes

The untreated Sn electrode produced a formic acid concentration of 170 mg L^−1^ under the selected operating conditions. Following electrochemical acid treatment, the concentration increased to 310 mg L^−1^, corresponding to an approximately 82% increase relative to untreated Sn. This improvement indicates that modification of the Sn surface positively influenced its electrochemical performance.

The enhanced performance of AtSn is consistent with the morphological changes observed during electrode characterization. The original electrode characterization showed that the untreated Sn surface was relatively smooth, whereas acid treatment produced a noticeably rougher surface. Such surface modification can increase the accessible electrode area and provide a more favorable surface for subsequent catalyst deposition. The treated surface also facilitated stabilization of the catalyst layer during electrode preparation. The beneficial role of structured Sn surfaces is also consistent with previous studies demonstrating that modification of Sn morphology and electrode architecture can improve CO_2_ reduction toward formate. Structured Sn electrodes have been reported to exhibit enhanced formate production compared with less-structured Sn surfaces [25].

#### 2.2.2. Effect of Bi–O–S Catalyst Deposition

Deposition of the Bi–O–S catalyst onto untreated Sn increased the formic acid concentration from 170 to 580 mg L^−1^, corresponding to an approximately 241% increase. This improvement highlights the contribution of the Bi-containing catalyst to CO_2_ reduction, consistent with the reported activity and formate selectivity of Bi-based nanostructures and composite catalysts [26,27,28,29]. The enhanced performance of Bi-based catalysts has been linked to their morphology, exposed active sites, and electronic environment. For example, nanostructured Bi nanoflakes have shown improved formate production owing to favorable edge and corner sites, while Bi nanoparticles supported on nitrogen-doped porous carbon have exhibited enhanced activity, emphasizing the role of catalyst–support interactions [28,29].

#### 2.2.3. Combined Effect of Substrate Treatment and Catalyst Incorporation

The highest formic acid concentration (750 mg L^−1^) was obtained with the Bi–O–S/AtSn electrode, compared with 170, 310, and 580 mg L^−1^ for untreated Sn, AtSn, and Bi–O–S/Sn, respectively. Thus, Bi–O–S/AtSn achieved approximately 4.4-fold higher formic acid concentration than untreated Sn under identical conditions. The progressive increase from 170 → 310 → 580 → 750 mg L^−1^ demonstrates the complementary contributions of Sn surface modification and Bi–O–S catalyst deposition. The enhanced performance can be attributed to the combination of the roughened acid-treated Sn surface, which facilitates catalyst attachment and interfacial contact, and the Bi–O–S layer, which provides the catalytically active phase. The FESEM/EDS results further confirm the modified surface morphology and successful distribution of Bi, O, and S across the catalyst layer.

This improvement is consistent with the broader role of electrode/interface engineering, where substrate architecture and catalyst–electrode interactions can influence catalyst utilization and CO_2_ reduction performance. Previous studies on structured Sn electrodes and Bi-containing catalysts have likewise demonstrated that controlling catalyst morphology and electrode architecture can enhance CO_2_ conversion toward formate [25,26,27,28,29].

### 2.3. Experimental Relationship Between Operating Conditions and CO_2_-to-Formic-Acid Performance

Following selection of the Bi–O–S/AtSn electrode, the effects of the principal operating variables on CO_2_ electroreduction were investigated using a 20-run central composite design (CCD). The three independent variables were applied current, catholyte concentration, and catalyst loading, investigated over ranges of 3–87 mA, 0.03–0.87 M, and 2.0–7.0 mg, respectively. The corresponding experimental responses were formic acid concentration and FE (FE). The experimental matrix demonstrates that the electrochemical response was strongly dependent on the applied operating conditions. Formic acid concentration varied from 49 to 948 mg L^−1^, whereas the measured FE ranged from 27 to 78% across the 20 experiments. These variations indicate that the three operating parameters exerted distinct effects on CO_2_ conversion and product selectivity. Importantly, the conditions that favored high formic acid concentration were not necessarily those producing the highest FE, establishing the productivity–selectivity relationship that is considered later in this section. The energy performance of the Bi–O–S/AtSn system was further evaluated in terms of specific energy consumption and energy efficiency, as summarized in Table 3. The specific energy consumption ranged from 3.02 to 8.52 kWh kg^−1^ HCOOH, while the corresponding energy efficiency varied from 18 to 51% across the 20 experimental runs.

#### 2.3.1. Effect of Applied Current

Applied current was a major factor governing formic acid production. At 0.45 M catholyte concentration and 4.5 mg catalyst loading, formic acid concentration increased markedly with current, from 49 mg L^−1^ at 3 mA to 538–750 mg L^−1^ around 45 mA and 863 mg L^−1^ at 87 mA. This trend reflects the increased electron flux at higher current, which promotes CO_2_ conversion and product accumulation. Similar current-dependent behavior has been reported for Sn-based CO_2_ reduction systems, where applied current and electrode structure influence reaction rate and selectivity [30,31]. In contrast, FE did not increase continuously with current. Under the central conditions, FE was approximately 76% at 3 mA, remained around 73–78% at 45 mA, but decreased to 54% at 87 mA. Thus, higher current enhanced the absolute formic acid concentration but reduced the fraction of charge directed toward formic acid at excessive current. This decline may arise from increased competing HER, mass-transfer limitations, and changes in the local electrode environment, consistent with reported current-dependent selectivity in Sn-based CO_2_ reduction [31,32]. Therefore, the results demonstrate that maximum current is not necessarily optimal, and an appropriate operating range is required to balance formic acid productivity and FE.

#### 2.3.2. Effect of Catholyte Concentration

Catholyte concentration also affected CO_2_-to-formic-acid conversion, with its influence depending on current and catalyst loading. Across the CCD range of 0.03–0.87 M NaHCO_3_ (central level: 0.45 M), no simple monotonic trend was observed. At 45 mA and 4.5 mg catalyst, the formic acid concentration was 493 mg L^−1^ at 0.03 M NaHCO_3_, varied between 462 and 750 mg L^−1^ among the repeated center-point experiments at 0.45 M, and reached 557 mg L^−1^ at 0.87 M. This behavior may be associated with changes in ionic conductivity, interfacial mass transport, bicarbonate/carbonate equilibria, local pH, and dissolved CO_2_ availability. While increased electrolyte concentration can improve ionic transport and reduce ohmic losses, excessive concentration may alter the local reaction environment and CO_2_ transport. The role of bicarbonate electrolytes in CO_2_RR is therefore dependent on the catalyst and operating conditions rather than being a simple proton source [33,34].

An additional consideration is the possible contribution of bicarbonate to the carbon-speciation and reaction environment during CO_2_ electroreduction. NaHCO_3_ participates in the dynamic CO_2_/HCO_3_^−^/CO_3_^2−^ equilibrium and can therefore influence dissolved inorganic carbon availability, local pH, CO_2_ transport, and interfacial reaction conditions [33]. Bicarbonate-derived species may also participate in coupled acid–base and interfacial reactions under electrochemical conditions [34]. Therefore, the measured formic acid concentration should be considered as the net product obtained under the CO_2_–bicarbonate electrolyte environment rather than being assigned exclusively to a single reaction pathway.

The interaction with current is further evident from the experimental data. At 20 mA and 3 mg catalyst, increasing NaHCO_3_ from 0.20 to 0.70 M increased formic acid concentration from 260 to 300 mg L^−1^ and FE from 61 to 70%. In contrast, at 70 mA, the same increase reduced formic acid concentration from 525 to 411 mg L^−1^ and FE from 35 to 27%. These contrasting responses demonstrate that catholyte concentration cannot be optimized independently and should instead be interpreted through its interaction with current, catalyst loading, and the local electrolyte environment.

#### 2.3.3. Effect of Catalyst Loading

Catalyst loading varied from 2.0 to 7.0 mg, with 4.5 mg as the central level. Its effect on formic acid production and FE depended on the applied current and catholyte concentration. At 20 mA, increasing the loading from 3 to 6 mg increased formic acid concentration from 260–300 to 312–317 mg L^−1^, while FE increased from 61–70% to 73–74%. This suggests that a higher catalyst loading can provide additional active sites and improve charge utilization under relatively low-current conditions. At 70 mA, the effect was more pronounced: increasing catalyst loading from 3 to 6 mg raised formic acid concentration from 411–525 to 842–948 mg L^−1^, depending on catholyte concentration. Thus, higher catalyst loading becomes particularly beneficial when the electron supply is sufficient to utilize the additional catalytic sites. However, excessive catalyst loading may produce thicker or more compact layers, promoting agglomeration, limiting electrolyte penetration, and increasing internal diffusion distances.

The observed response therefore represents a balance between active-site availability and mass transport, consistent with reported effects of catalyst structure and loading in Bi- and Sn-based CO_2_RR systems [35,36]. The highest formic acid concentration in the CCD experiments, 948 mg L^−1^, was obtained at 70 mA, 0.20 M catholyte, and 6 mg catalyst, while 842 mg L^−1^ was obtained at the same current and catalyst loading with 0.70 M catholyte. These results further confirm that catalyst loading must be optimized together with the other process variables rather than considered independently.

### 2.4. Response Surface Modeling and Process Optimization

The CCD results were subsequently analyzed using RSM to quantify the interactions among applied current, catholyte concentration, and catalyst loading. Because formic acid concentration and FE exhibited different response trends, the two responses were analyzed separately before simultaneous optimization. The experimental domain comprised applied currents of 3–87 mA, catholyte concentrations of 0.03–0.87 M, and catalyst loadings of 2–7 mg. The corresponding experimental responses covered a broad range, with formic acid concentration varying from 49 to 948 mg L^−1^ and FE from 27 to 78% across the 20 CCD experiments. The response-surface analysis was therefore used to identify the operating regions associated with enhanced formic acid production and improved selectivity. Because the two responses do not exhibit identical trends with changing operating conditions, they were evaluated separately before being considered simultaneously during the subsequent multi-objective optimization.

#### 2.4.1. Central Composite Design and Experimental Domain

The CCD was constructed using applied current, catholyte concentration, and catalyst loading as the three independent variables. The experimental design included factorial, axial, and center-point conditions within the selected operating domain. The central experimental condition was 45 mA, 0.45 M catholyte, and 4.5 mg catalyst, while the experimental boundaries were 3–87 mA, 0.03–0.87 M, and 2–7 mg, respectively. The experimental results demonstrated substantial changes in both responses across this domain. Increasing the applied current produced a strong increase in formic acid concentration, whereas FE generally showed the opposite tendency at higher current. The catalyst loading and catholyte concentration modified these responses through their interaction with the applied current. Such coupled behavior justifies the use of response-surface analysis rather than relying exclusively on one-factor-at-a-time interpretation. The importance of considering these variables simultaneously has also been recognized in CO_2_ electrolysis studies, where current density, electrolyte environment, catalyst architecture, and reactor conditions collectively influence productivity and selectivity [37,38].

#### 2.4.2. Response Surface for Formic-Acid Concentration

**Figure 2:**
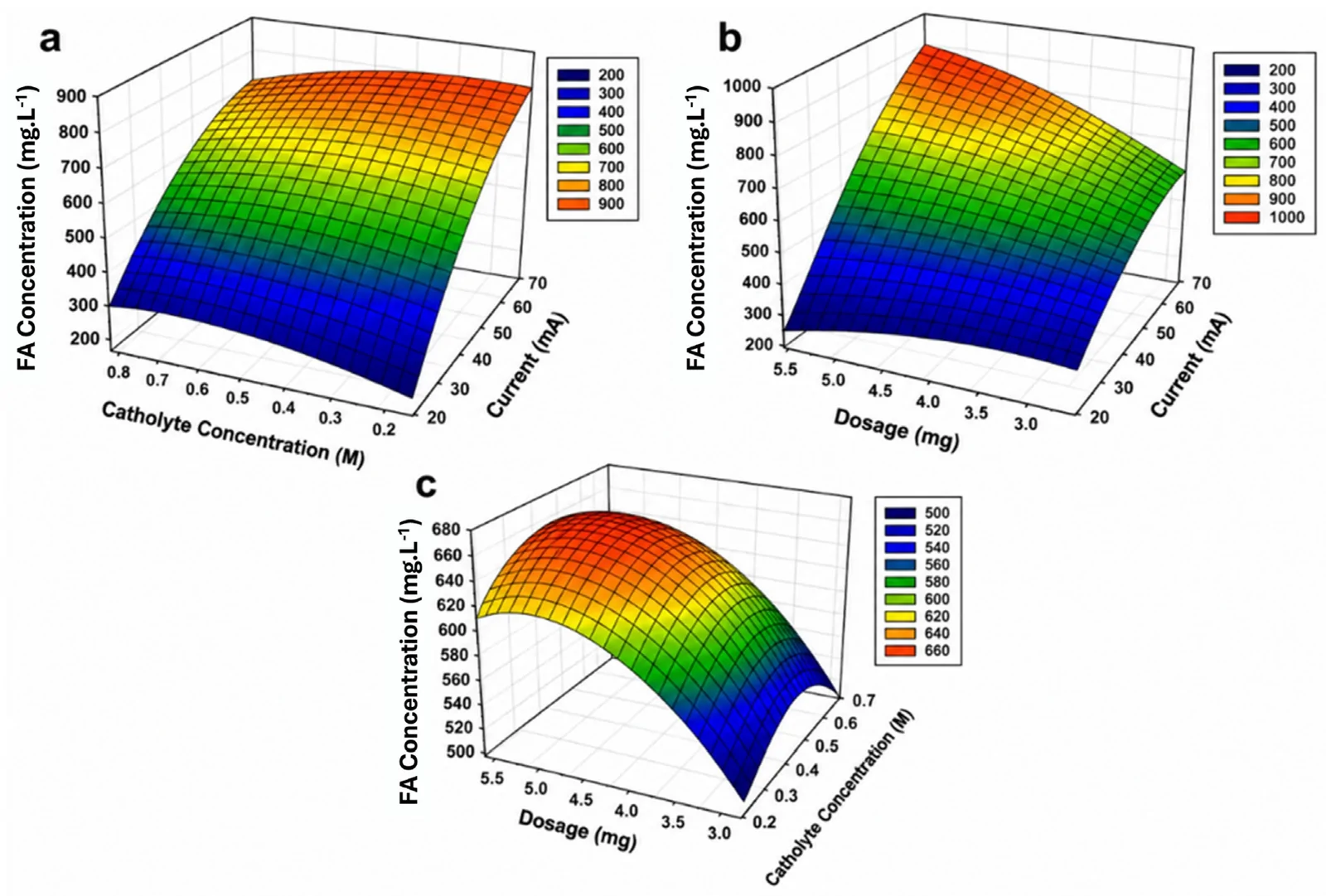
Three-dimensional response surface plots illustrating the interactive effects of (**a**) catholyte concentration and applied current, (**b**) catalyst dosage and applied current, and (**c**) catalyst dosage and catholyte concentration on formic acid concentration during the electrochemical reduction in CO_2_.

The response surfaces for formic acid concentration demonstrate that the applied current was the principal variable controlling the magnitude of product formation within the investigated domain. The highest region of the response surface occurred at relatively high current combined with intermediate-to-moderate catholyte concentration and increased catalyst loading. The response surface generated from the original experimental dataset shows (Figure 2) that formic acid concentration approached approximately 900–1000 mg L^−1^ in the high-current region.

##### Current Density–Catholyte Interaction

The interaction between applied current and catholyte concentration was evaluated at a fixed catalyst loading of 4.5 mg. The response surface shows a pronounced increase in formic acid concentration with increasing current. At lower currents, the response remained comparatively low across the catholyte concentration range, whereas substantially higher concentrations were obtained as the current approached the upper region of the experimental domain. The highest response region occurred at an applied current of approximately 70 mA and a catholyte concentration around 0.35 M.

The current dependence reflects the greater electron flux available for electrochemical CO_2_ conversion at higher current. However, the response does not increase indefinitely with catholyte concentration, indicating that electrolyte concentration provides a secondary control over the reaction environment. An intermediate catholyte concentration appears favorable within the investigated domain, where ionic transport and CO_2_ availability can be balanced. This interaction is important because it demonstrates that the effect of catholyte concentration depends on the applied current. Therefore, the catholyte concentration cannot be considered as an independent optimum without simultaneously specifying the current condition.

##### Current Density–Catalyst Loading Interaction

The current–catalyst interaction was evaluated at a catholyte concentration of 0.45 M. The response surface indicates that increasing current substantially enhances formic acid concentration, while increasing catalyst loading provides an additional improvement within the investigated range. The highest response region approached 1000 mg L^−1^ at an applied current of approximately 70 mA and catalyst loading of approximately 6 mg. The positive influence of catalyst loading at elevated current can be associated with the availability of additional catalytic sites capable of utilizing the increased electron flux. However, the response surface also indicates that simply increasing catalyst loading does not provide an unlimited benefit. Excessive loading can produce a thicker catalyst layer and may introduce transport limitations or inefficient utilization of catalyst material. The interaction therefore reflects a balance between the electron supply imposed by the applied current and the number of accessible catalytic sites provided by the catalyst layer. This type of catalyst–operating-condition interaction is consistent with the importance of catalyst architecture and interfacial engineering in Bi- and Sn-based CO_2_ reduction systems [38,39,40].

##### Catholyte Concentration–Catalyst Loading Interaction

The combined influence of catholyte concentration and catalyst loading was evaluated at a fixed current of 45 mA. In this case, the response surface exhibited a comparatively broader high-response region than that observed for the current-dependent interactions. The maximum formic acid concentration was approximately 680 mg L^−1^ at a catalyst loading of around 5.5 mg and catholyte concentration close to 0.35 M. At catalyst loadings below approximately 4 mg, the response remained comparatively limited over the investigated catholyte concentration range. This suggests that insufficient catalyst availability can restrict the conversion response under these conditions. Increasing the catalyst loading provides additional active sites, while an appropriate catholyte concentration supports ionic transport and the electrochemical reaction environment.

#### 2.4.3. Response Surface for FE

**Figure 3:**
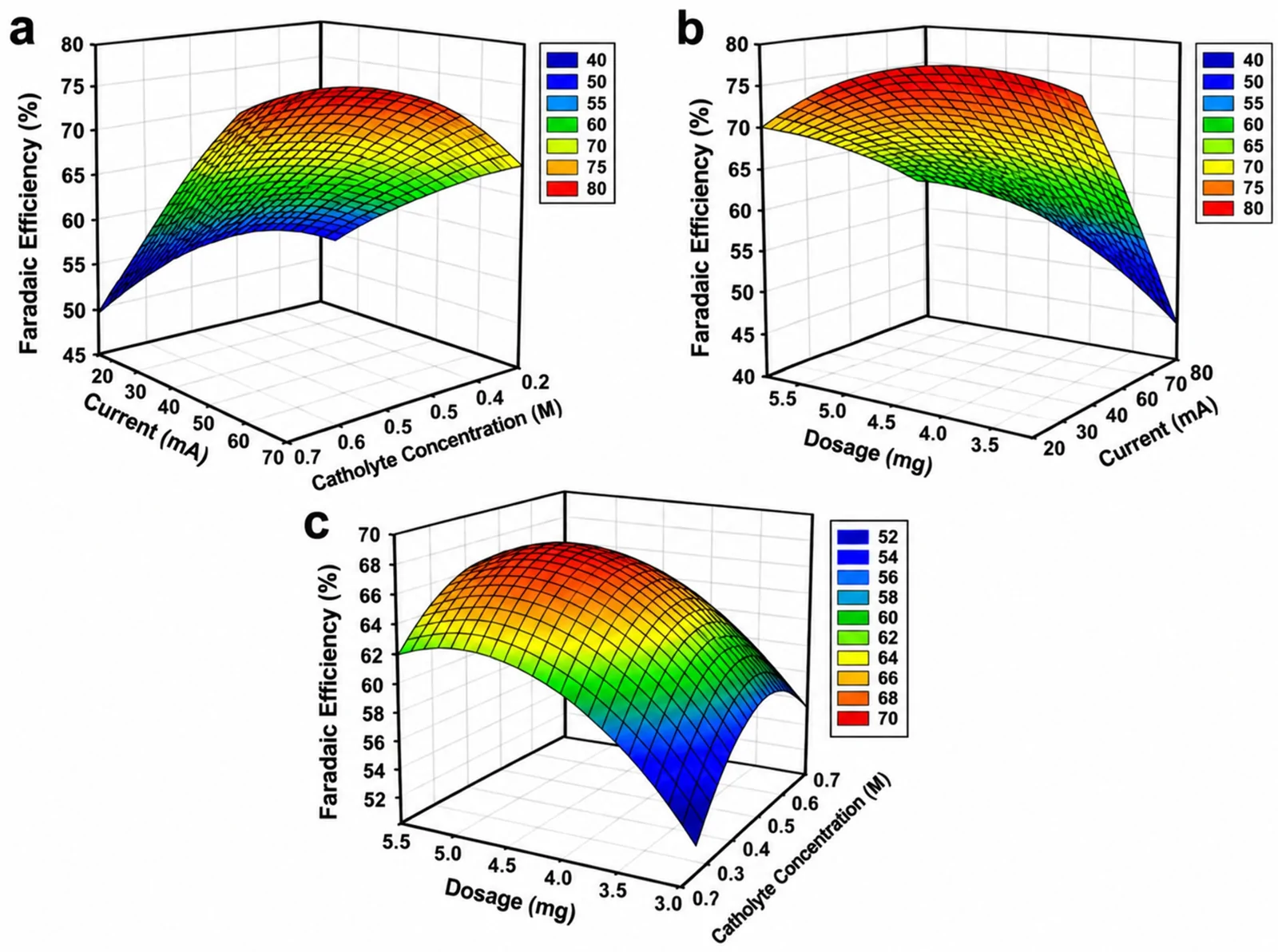
Three-dimensional response surface plots illustrating the interactive effects of (**a**) catholyte concentration and applied current, (**b**) catalyst dosage and applied current, and (**c**) catalyst dosage and catholyte concentration on FE during the electrochemical reduction in CO_2_.

The response surfaces for FE (Figure 3) exhibited trends that differed from those observed for formic-acid concentration. Whereas increasing current generally enhanced the absolute formic-acid concentration, FE tended to decline as the current increased. The highest FE region was therefore associated with lower-to-intermediate current conditions rather than the high-current region that favored maximum product concentration.

##### Current Density–Catholyte Interaction

The response surface for FE at a fixed catalyst loading of 4.5 mg shows a strong dependence on applied current. The highest FE region was obtained at relatively low current and intermediate catholyte concentration, with the maximum region approaching 80% FE around 20 mA and approximately 0.35 M catholyte. In contrast to formic-acid concentration, increasing the current beyond this region resulted in lower FE. This behavior indicates that the additional electron supply at high current does not translate proportionally into formic acid formation. A greater fraction of the charge can instead be associated with competing processes, including HER, together with changes in the local reaction environment and transport conditions. Thus, the current–catholyte interaction provides direct evidence of the fundamental distinction between product concentration and Faradaic selectivity. Conditions favorable for rapid product accumulation are not necessarily those producing the highest fraction of charge directed toward formic acid.

##### Current Density–Catalyst Loading Interaction

The current–catalyst loading response surface was evaluated at a catholyte concentration of 0.45 M. The highest FE region occurred at relatively low current and moderate catalyst loading, with an optimum region of approximately 80% FE around 20 mA and 4.5 mg catalyst. Although increasing catalyst loading can increase the number of available active sites, the response surface indicates that applied current exerts a stronger influence on FE within the investigated domain. At elevated current, the decrease in FE becomes more pronounced even when the catalyst loading is increased. This behavior further emphasizes that increasing catalyst loading cannot completely compensate for the selectivity loss associated with excessive current. The catalyst loading therefore acts as a secondary parameter that can modify the response, whereas control of current is essential for maintaining high formic-acid selectivity.

##### Catholyte Concentration–Catalyst Loading Interaction

The interaction between catholyte concentration and catalyst loading was evaluated at a fixed applied current of 45 mA. The response surface indicates that moderate catalyst loading combined with an intermediate catholyte concentration favored higher FE. The highest region was approximately 70% FE, occurring around 5 mg catalyst and 0.45 M catholyte. The observed response can be associated with the combined influence of catalyst availability and the electrolyte environment. Adequate catalyst loading provides sufficient accessible active sites, while an appropriate catholyte concentration supports ionic transport and the interfacial electrochemical environment. However, compared with the strong influence of applied current, this interaction produced a more moderate effect on FE.

### 2.5. Multi-Objective Optimization and Experimental Validation

The response-surface analysis demonstrated that the operating conditions producing the highest formic-acid concentration were different from those favoring the highest FE. Therefore, simultaneous optimization was performed to identify a practical operating condition that balances product concentration and selectivity. Numerical optimization of the RSM model was used to evaluate the individual response maxima and subsequently identify a compromise operating condition for the two responses. Such multi-objective optimization is particularly relevant for electrochemical CO_2_ conversion because maximizing product formation and maximizing Faradaic selectivity can require different operating conditions [40,41,42,43,44]. The optimization results and experimental validation are summarized in Table 4, while the complete RSM-predicted and experimentally measured responses are provided in Supplementary Table S1; the corresponding parity plots and Minitab optimization plot are presented in Supplementary Figures S1–S3.

#### 2.5.1. Optimization of Formic-Acid Concentration

When formic acid concentration was treated as the sole optimization objective, the RSM model predicted a maximum concentration of 1082 mg L^−1^ at an applied current of 87 mA, catholyte concentration of 0.26 M, and catalyst loading of 7.0 mg. This result is consistent with the response-surface behavior, where increased applied current and higher catalyst loading favored greater formic-acid accumulation. The need to balance production rate with energy efficiency and product selectivity has similarly been emphasized in techno-economic assessments of electrochemical CO_2_ conversion systems [45,46]. The predicted maximum represents the productivity-oriented boundary of the investigated operating domain rather than the overall optimum for both performance criteria. Because this condition was obtained by maximizing formic-acid concentration alone, it does not necessarily correspond to the highest FE. Therefore, it was not selected as the final operating condition for simultaneous optimization.

#### 2.5.2. Optimization of FE

When FE was used as the sole optimization objective, the model predicted a maximum FE of 85% at an applied current of 3 mA, catholyte concentration of 0.63 M, and catalyst loading of 3.4 mg. The substantially lower current associated with the FE optimum is consistent with the experimental and response-surface trends described previously. This distinction between productivity and selectivity is an important consideration in the design and optimization of electrochemical CO_2_ reduction systems [47,48,49]. Lower current reduces the contribution of competing electrochemical pathways and therefore favors charge utilization toward formic-acid formation. However, this condition is associated with substantially lower formic-acid production than the productivity-oriented operating region. Consequently, maximizing FE alone would not provide the desired balance between product concentration and electrochemical selectivity.

#### 2.5.3. Compromise Optimization of Productivity and Selectivity

Because the individual response maxima occurred at different operating conditions, simultaneous numerical optimization was performed to obtain a compromise between formic-acid concentration and FE. The RSM model identified an applied current of 73 mA, catholyte concentration of 0.33 M, and catalyst loading of 6.5 mg as the compromise operating condition. Under these conditions, the model predicted a formic acid concentration of 950 mg L^−1^ and an FE of 61%. This compromise condition is particularly important because it does not represent the mathematical maximum of either response individually. Instead, it provides a balanced operating point in which a high formic-acid concentration is retained while maintaining a moderate FE. The selected condition therefore represents the principal process optimum of the study and was used for experimental confirmation.

#### 2.5.4. Experimental Validation of the Predicted Optimum

A confirmation experiment was performed using the operating conditions predicted by simultaneous optimization: 73 mA applied current, 0.33 M catholyte concentration, and 6.5 mg catalyst loading. The experimental formic acid concentration was 1020 mg L^−1^, compared with the RSM prediction of 950 mg L^−1^. Similarly, the experimentally measured FE was 67%, compared with the predicted value of 61%. Thus, the experimental confirmation produced higher responses than the corresponding model predictions while remaining within a reasonable deviation range. The experimental values also confirm that the compromise operating condition identified through numerical optimization was experimentally achievable.

#### 2.5.5. Prediction Deviation and Model Performance

The deviation between the predicted and experimentally measured responses was calculated relative to the predicted value. Accordingly, the experimental values were 7.37% higher than the predicted formic-acid concentration and 9.84% higher than the predicted FE. The relatively small deviations indicate reasonable agreement between the RSM predictions and the confirmation experiment. The source data similarly identify the experimental values of 1020 mg L^−1^ and 67% against predictions of 950 mg L^−1^ and 61%, respectively. The validation therefore supports the use of the developed RSM framework for identifying an operating region that balances formic-acid production and FE. The validation represents confirmation at the selected optimum rather than an independent demonstration of model validity across the entire experimental domain.

### 2.6. Derived Productivity and Electrocatalytic Performance Indicators

The validated optimum delivered 1020 mg L^−1^ formic acid at 67% FE under 73 mA, 0.33 M catholyte, and 6.5 mg catalyst. The corresponding formic acid production rate was 40.8 mg h^−1^, equivalent to a molar production rate of 0.887 mmol h^−1^. Based on the 1 cm^2^ electrode area, the formic-acid partial current density was 48.9 mA cm^−2^, while the catalyst-normalized productivity was 6.28 mg h^−1^ mg_cat^−1^. These metrics provide complementary measures of absolute productivity, charge utilization, and catalyst utilization and therefore extend the performance assessment beyond concentration and FE alone.

### 2.7. Electrochemical Stability and Performance Retention

**Figure 4:**
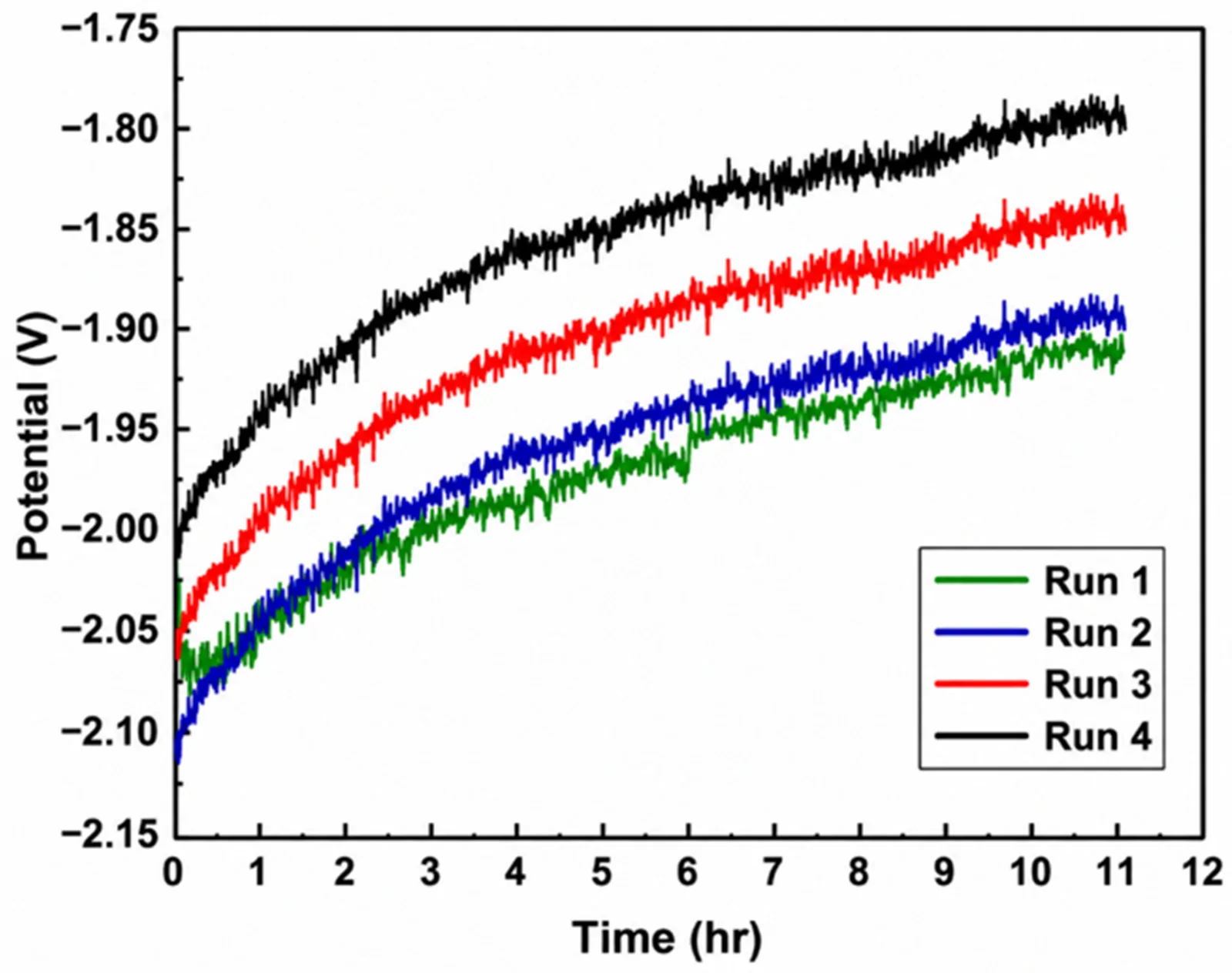
Chronopotentiometric stability profiles of the Bi–O–S/acid-treated Sn electrode under the optimized operating conditions.

Long-term stability was evaluated under the optimized conditions of 73 mA applied current, 0.33 M catholyte concentration, and 6.5 mg catalyst loading. Long-term electrode stability is an important consideration for the practical implementation of electrochemical CO_2_ conversion because catalyst lifetime can substantially influence overall process performance [50]. The electrode exhibited relatively stable chronopotentiometric behavior (Figure 4) during the initial period of prolonged electrolysis, followed by a noticeable increase in operating potential after approximately 11 h, indicating gradual deterioration in electrochemical performance.

The consecutive 11 h experiments (Table 5) showed that formic acid production remained relatively consistent during the first three cycles, yielding 12,800, 12,700, and 12,650 mg L^−1^, with corresponding FEs of 74, 74, and 73%, respectively. The corresponding formic acid production rates were 46.5, 46.2, and 46.0 mg h^−1^, demonstrating close agreement among the first three cycles. Thus, the electrode retained comparable productivity and selectivity over the first 33 h of cumulative operation when fresh catholyte was used between cycles. The formic acid concentration obtained during the long-term experiment can be correlated with the electrolysis duration and the product accumulation in the catholyte. Under the optimized conditions, the 1 h validation experiment in Table 4 yielded 1020 mg L^−1^ formic acid, corresponding to a production rate of 40.8 mg h^−1^ based on the 40 mL catholyte volume. In comparison, the 12,800 mg L^−1^ obtained in Cycle 1 of Table 5 represents the accumulation of formic acid over 11 h, corresponding to approximately 512 mg of formic acid in the 40 mL catholyte and an average production rate of 46.5 mg h^−1^. On a time-normalized basis, the Cycle 1 concentration corresponds to approximately 1164 mg L^−1^ h^−1^, compared with 1020 mg L^−1^ h^−1^ in the 1 h validation experiment. The similar magnitude of these values indicates that the higher absolute formic acid concentration observed in Table 5 is predominantly associated with the extended electrolysis duration and cumulative product formation.

The sustained product accumulation during the 11 h stability experiments were also associated with continuous CO_2_ availability during electrolysis. Although the catholyte was initially saturated with CO_2_, continuous CO_2_ supply maintained the availability of CO_2_ for electrochemical conversion throughout the experiment. Consequently, formic acid concentration increased progressively with electrolysis time rather than being restricted to the initial dissolved CO_2_ inventory. The higher concentration measured after 11 h therefore reflects extended product accumulation under sustained CO_2_ availability. The slightly higher average production rate observed in Cycle 1 (46.5 mg h^−1^) relative to the 1 h validation experiment (40.8 mg h^−1^) was accompanied by a corresponding increase in FE from 67% to 74% and may reflect normal variation between individual electrochemical experiments.

The first three stability cycles production rates of 46.5, 46.2, and 46.0 mg h^−1^ corresponded to FEs of 74, 74, and 73%, respectively, indicating consistent conversion of CO_2_ to formic acid during the initial 33 h of cumulative operation. The use of fresh catholyte between cycles further indicates that the concentrations reported for each cycle correspond to product accumulated during the respective 11 h electrolysis period rather than cumulative accumulation in a single electrolyte over the entire stability test. A pronounced decline was observed in the fourth cycle, where the formic acid concentration decreased to 8500 mg L^−1^, corresponding to an average production rate of 30.9 mg h^−1^, while the FE decreased to 49%. Relative to the first cycle, these values correspond to decreases of approximately 33.6% in formic acid concentration, 33.5% in production rate, and 33.8% in FE, respectively, and this reduction can be attributed due to catalyst instability during the fourth cycle. The simultaneous decrease in production rate and FE indicates progressive deterioration during prolonged operation. This deterioration may arise from catalyst/electrode deactivation and/or changes in electrolyte composition during extended electrolysis.

### 2.8. Benchmarking Against Previously Reported Bi-Based Electrocatalysts

The performance of the Bi–O–S/acid-treated Sn electrode was compared (Table 6) with representative Bi-based electrocatalysts reported for electrochemical CO_2_ conversion to formate/formic acid. The comparison considers catalyst/electrode configuration, electrolyte, current density, FE, productivity, and stability, where available [51,52]. As summarized in Table 6, the Bi–O–S/acid-treated Sn electrode developed in this work exhibits a favorable combination of current density, formate productivity, and operational stability compared with representative Bi-based electrocatalysts reported previously. Although several reported catalysts achieve higher formate Faradaic efficiencies (84–95%), these high FE values were generally obtained at relatively low current densities (5–30 mA cm^−2^), and the corresponding formate/formic acid production rates were not reported (NR). In contrast, the present electrode achieves a substantially higher current density of 73 mA cm^−2^, together with a quantified formate production rate of 1020 mg L^−1^ min^−1^ (40.8 mg h^−1^), while maintaining a formate FE of 67%. Moreover, the electrode exhibits stable operation for 33 h, which exceeds the 10–12 h stability reported for the comparative systems for which stability data are available. These results demonstrate that the present system provides a more balanced combination of high reaction rate, measurable formate productivity, and long-term operational stability.

Although the Bi–O–S/acid-treated Sn electrode showed lower FE than several reported Bi-based catalysts, which often achieve FE values above 90%, the present system was optimized for both productivity and selectivity rather than FE alone. The Bi–O–S/acid-treated Sn electrode exhibited a formic acid concentration of 1020 mg L^−1^ and a production rate of 40.8 mg h^−1^ at 67% FE under the optimized conditions, while relatively stable performance was retained over the first three consecutive 11 h cycles (33 h cumulative operation), followed by a decrease in performance during the fourth cycle. The progressive increase in formic acid concentration from 170 mg L^−1^ (untreated Sn) → 310 mg L^−1^ (AtSn) → 580 mg L^−1^ (Bi–O–S/Sn) → 750 mg L^−1^ (Bi–O–S/AtSn) further demonstrates the combined contribution of substrate engineering and Bi–O–S incorporation. The principal advantage of the Bi–O–S/AtSn architecture lies in its integrated catalyst–substrate design and balanced productivity–selectivity, rather than maximum FE alone. The experimentally validated production rate of 40.8 mg h^−1^, together with the optimized formic acid concentration and 33 h performance retention, provides a basis for further development and stability improvement in this electrode.

### 2.9. Proposed Interpretation of the Bi–O–S/Sn Catalytic System

#### 2.9.1. Role of the Bi-Containing Phase

Bismuth-based materials are widely recognized as selective electrocatalysts for CO_2_ reduction to formate because their surface properties favor oxygen-bound intermediates associated with the formate pathway. Stabilization of OCHO-type intermediates has been proposed as an important step in CO_2_-to-formate conversion [52]. Recent studies further demonstrate that phase composition, surface structure and electronic modification of Bi can significantly influence its activity and selectivity [49].

The Bi–O–S catalyst characterized in this study contains Bi, O and S species distributed throughout the catalyst layer. The presence of these components may provide chemically distinct surface environments that influence CO_2_ adsorption and stabilization of oxygen-containing intermediates. Accordingly, the Bi-containing phase is proposed to provide the principal catalytic sites for CO_2_ conversion, while the O- and S-containing species may modify the local electronic and interfacial environment. This interpretation is consistent with reported strategies involving compositional and surface modification of Bi-based catalysts [49,52].

#### 2.9.2. Role of the Acid-Engineered Sn Substrate

The improvement obtained after acid treatment of the Sn substrate indicates that the substrate itself contributes to the overall electrochemical response. Sn is intrinsically active toward CO_2_-to-formate conversion, with mechanistic studies identifying OCHO as a favorable intermediate during CO_2_ reduction on Sn [47]. The subsequent incorporation of Bi–O–S onto the acid-treated Sn surface produced a further enhancement in formic acid formation, suggesting that the catalyst–substrate interface plays an important role in the observed performance. The acid-engineered Sn substrate may therefore serve both as a conductive support and as an additional catalytically relevant component. The enhanced behavior is consequently interpreted as arising from the combined Bi–O–S/Sn interfacial system, rather than from the Bi-containing phase alone. Interface and compositional engineering are recognized strategies for modifying the activity of Sn- and Bi-based formate-producing catalysts [45].

#### 2.9.3. Relationship Between Current Density, Productivity and HER

Increasing the applied current increases the electron-transfer rate and can consequently enhance the rate of formic acid formation. However, higher cathodic current can also increase the contribution of competing hydrogen evolution, while CO_2_ availability at the electrode surface may become increasingly limiting [21,49]. The observed productivity–selectivity relationship is therefore consistent with competition between CO_2_ reduction and HER. Recent mechanistic modeling of CO_2_ reduction on Sn explicitly considers the simultaneous occurrence of CO_2_ reduction and HER and identifies OCHO as a key intermediate in the formate pathway [49]. The optimized operating condition can consequently be regarded as a compromise between maintaining sufficient electron flux for CO_2_ conversion and limiting competing hydrogen evolution.

#### 2.9.4. Proposed CO_2_-to-Formic-Acid Reaction Pathway

**Figure 5:**
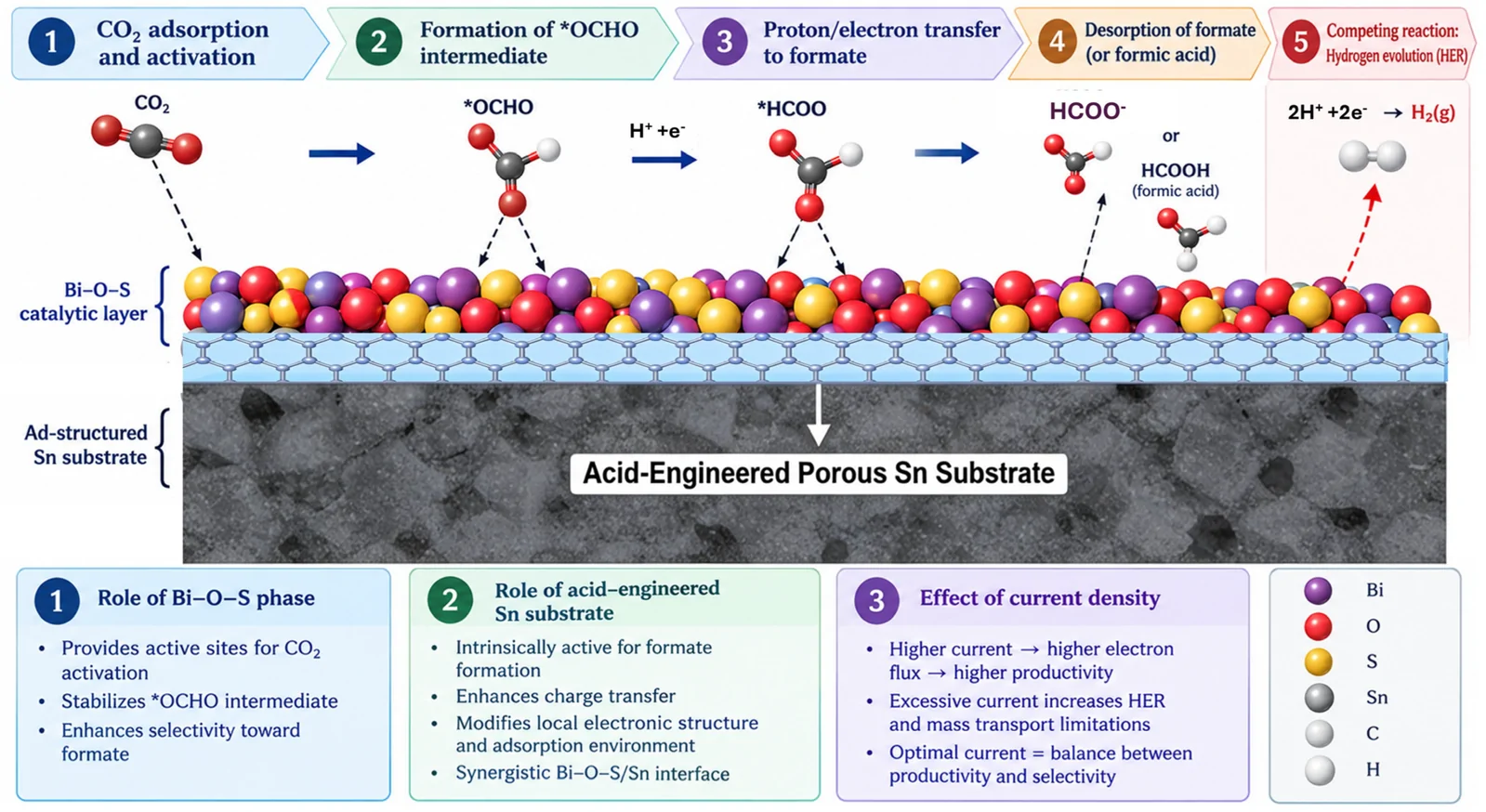
Proposed literature-supported CO_2_-to-formate pathways and interfacial role of the Bi–O–S/acid-treated Sn electrode. These reaction pathways and intermediates are only schematic interpretations.

Based on established mechanisms for Bi- and Sn-based CO_2_ reduction, a plausible pathway (Figure 5) involves adsorption and activation of CO_2_ followed by proton-coupled electron transfer to form an oxygen-bound OCHO intermediate. Further proton/electron transfer leads to formate, which can exist in equilibrium with formic acid depending on the local protonation environment [41,47].

CO_2_ + H^+^ + 2e^−^ → OCHO → HCOO^−^/HCOOH

The Bi-containing phase is proposed to facilitate the CO_2_-to-OCHO pathway, while the acid-engineered Sn substrate may influence charge transfer and the local interfacial environment. Simultaneously, HER competes for electrons and protons, particularly under conditions of increased cathodic driving force [31,47].

## 3. Materials and Methods

### 3.1. Chemicals and Materials

All chemicals used in this study were of analytical grade and were used as received without further purification. Bismuth nitrate pentahydrate [Bi(NO_3_)_3_·5H_2_O], sodium sulfate (Na_2_SO_4_), potassium hydroxide (KOH), sodium bicarbonate (NaHCO_3_), sulfuric acid (H_2_SO_4_), ethanol (C_2_H_5_OH), acetone (C_3_H_6_O), Nafion^®^ D-521 dispersion, and Nafion^®^ 117 membrane were purchased from Sigma-Aldrich, St. Louis, Missouri, USA. Tin (Sn) foil with a purity of 99.95% and a thickness of 0.5 mm was used as the electrode substrate. Deionized (DI) water was used for the preparation of all aqueous solutions and for electrode washing. High-purity CO_2_ (99.99%) was used as the reactant gas during the electrochemical reduction experiments.

### 3.2. Synthesis of the Bismuth-Based Bi–O–S Catalyst

The Bi–O–S catalyst was synthesized by a precipitation-assisted method using Bi(NO_3_)_3_·5H_2_O and Na_2_SO_4_ as precursor materials. Briefly, 20 mL of 1 M Bi(NO_3_)_3_·5H_2_O and 20 mL of 1 M Na_2_SO_4_ solutions were mixed under continuous magnetic stirring at room temperature, followed by dropwise addition of 2 mL of 1M H_2_SO_4_. The mixture was stirred for 30 min to promote homogeneous precipitation and subsequently ultrasonicated for 60 min to improve particle dispersion. After standing for 30 min, the precipitate was collected and thoroughly washed several times with ethanol and deionized water to remove residual soluble species. The washed precipitate was then dried at 110 °C for 2 h followed by calcination at 500 °C for 2 h in a muffle furnace. The resulting Bi–O–S catalyst was stored in an airtight glass vial until characterization and electrode fabrication.

### 3.3. Electrochemical Acid Treatment of the Sn Substrate

Tin foil was cut into 1 × 1 cm^2^ pieces and initially rinsed with DI water to remove surface contaminants. The Sn substrates were electrochemically treated in 1 M H_2_SO_4_ using a potentiostat under a constant applied potential of 3 V for 40 s. Following electrochemical treatment, the substrates were immersed in a mixture of acetone and ethanol and ultrasonicated for 10 min to remove residual organic contaminants. The treated substrates were subsequently rinsed thoroughly with DI water and dried under ambient conditions prior to catalyst deposition. The electrochemically treated substrate is designated as acid-treated Sn (AtSn) throughout the manuscript. The geometric area exposed for subsequent electrochemical measurements was maintained at 1 cm^2^.

### 3.4. Fabrication of Bi–O–S/Acid-Treated Sn Electrodes

A catalyst ink was prepared by dispersing the required mass of the synthesized Bi–O–S catalyst in ethanol containing PTFE binder. The suspension was ultrasonicated for 2 h to obtain a homogeneous catalyst ink. The resulting ink was uniformly deposited onto the electrochemically treated Sn substrate by drop-casting using a micropipette. The coated electrodes were dried at 80 °C for 2.5 h to remove the solvent and promote adhesion and stabilization of the PTFE-bound catalyst layer. The geometric electrode area was maintained at 1 cm^2^. Electrodes with different catalyst dosages were prepared using the same fabrication procedure for the subsequent electrochemical experiments and statistical optimization. The resulting catalyst-coated electrode is referred to as the Bi–O–S/AtSn electrode.

### 3.5. Physicochemical Characterization

The structural, chemical, morphological, and textural properties of the Bi–O–S catalyst and Bi–O–S/acid-engineered Sn electrodes were characterized using complementary techniques. X-ray diffraction (XRD) was performed using a Bruker D8 Advance diffractometer (Bruker AXS GmbH, Karlsruhe–Knielingen, Germany) with Cu Kα radiation (λ = 1.5406 Å) at 40 kV and 40 mA. Surface chemical states and elemental composition were analyzed by X-ray photoelectron spectroscopy (XPS; AXIS Ultra DLD, Kratos Analytical Ltd., Manchester, UK), with emphasis on Bi, O, and S species. Surface morphology and elemental distribution were examined by field-emission scanning electron microscopy (FESEM; Nova NanoSEM 450, Thermo Fisher Scientific, Waltham, MA, USA) operated at 10 kV, coupled with EDS/EDX and elemental mapping. Transmission electron microscopy (TEM) was employed to resolve the nanoscale morphology and structural features of the Bi–O–S catalyst. Nitrogen adsorption–desorption measurements were conducted using a TriStar II analyzer (Micromeritics, Norcross, GA, USA) to determine the BET-specific surface area and pore characteristics.

### 3.6. Electrochemical CO_2_ Reduction Experiments

Electrochemical CO_2_ reduction experiments were performed using a Corrtest electrochemical workstation (CS21500,Wuhan Corrtest Instruments Corp., Ltd., Wuhan, China) in a two-compartment H-type cell separated by a Nafion^®^ 117 proton-exchange membrane (Chemours Company, Wilmington, DE, USA). The Bi–O–S/AtSn electrode, Ag/AgCl electrode, and Pt wire were used as the working, reference, and counter electrodes, respectively. Each compartment contained 40 mL of electrolyte, with aqueous NaHCO_3_ solutions of varying concentrations used as the catholyte and 1 M KOH as the anolyte. Before electrolysis, both compartments were purged with N_2_ for 5 min, followed by CO_2_ saturation of the catholyte using high-purity CO_2_ (99.99%) at 10 mL min^−1^ for 30 min. CO_2_ was continuously supplied to the cathodic compartment during electrolysis. Experiments were conducted under galvanostatic conditions for 60 min, except for long-term stability tests. After each experiment, the catholyte was collected for liquid-product analysis.

### 3.7. HPLC Analysis and FE Calculation

Formic acid generated during CO_2_ electroreduction was quantified by high-performance liquid chromatography (HPLC; Shimadzu LC-2050C LT, Kyoto, Japan) equipped with a Shim-pack SCR-102H column and refractive index detector. A 0.5 mM HClO_4_ solution was used as the mobile phase. The formic acid concentration in the catholyte collected after electrolysis was determined from the corresponding calibration curve. The amount of formic acid produced (*n*_HCOOH_) (mol) was calculated from the measured concentration, (*C*_HCOOH_) (mg L^−1^), and catholyte volume (*V*) (L), according to Equation (1):

(1) $$\left[n_{\mathrm{HCOOH}}=\frac{C_{\mathrm{HCOOH}}V}{1000M_{\mathrm{HCOOH}}}\right]$$

where (*M*_HCOOH_) is the molar mass of formic acid (46.03 g mol^−1^) and the factor of 1000 converts mg to g. The FE for formic acid was calculated as in Equation (2):

(2) $$\left[FE_{\mathrm{HCOOH}}=\frac{n_{\mathrm{HCOOH}},\mathrm{zF}}{Q}\times 100\right]$$

where (*n*_HCOOH_) is the number of moles of formic acid produced, (*z*) is the number of electrons required for the formation of one mole of formic acid ((z = 2)), (F) is the Faraday constant (96,485 C mol^−1^), and (*Q*) is the total charge passed during electrolysis (C) [32].

### 3.8. Response Surface Methodology and Central Composite Design

Response surface methodology (RSM) coupled with a central composite design (CCD) was employed to evaluate the individual and interactive effects of key operating parameters on electrochemical CO_2_ conversion. A second-order response model was developed to describe linear, interaction, and quadratic effects while minimizing the experimental requirement. Three independent variables, namely applied current, NaHCO_3_ catholyte concentration, and catalyst dosage, were selected based on preliminary electrochemical experiments. Their experimental ranges were 3–87 mA, 0.03–0.87 M, and 2.0–7.0 mg, respectively, and were converted to coded levels for CCD construction. The design comprised 20 experimental runs generated using Minitab 19. The mean values were used for model development, with formic acid concentration and FE selected as the principal response variables. The experimental responses were fitted to a second-order polynomial model of the general form in Equation (3):

(3) $$\left[Y=\beta_{0}+\sum_{i=1}^{n}\beta_{i}X_{i}+\sum_{i=1}^{n}\beta_{\mathrm{ii}}X_{i}^{2}+\sum_{i<j}\beta_{\mathrm{ij}}X_{i}X_{j}\right]$$

where (Y) represents the predicted response, (*β*_0_) is the model intercept, (*β_i_*) represents the linear coefficient, (*β_ii_*) represents the quadratic coefficient, (*β_ij_*) represents the interaction coefficient, and (*X_i_*) and (*X_j_*) are the coded independent variables. The fitted models were used to evaluate the significance of the individual variables and their interactions and to generate response surfaces for the two selected responses. Numerical optimization was subsequently used to identify operating conditions providing a compromise between formic-acid production and FE.

### 3.9. Derived Electrochemical Performance Metrics

To provide a consistent assessment of electrode activity, productivity, and selectivity, complementary electrochemical performance metrics were calculated from the applied current, geometric electrode area, electrolysis time, catholyte volume, product concentration, and catalyst mass. The geometric current density was calculated as in Equation (4):

(4) $$\left[j=\frac{I}{A}\right]$$

where (j) is the geometric current density (mA cm^−2^), *(I*) is the applied current (mA), and (*A*) is the geometric electrode area (cm^2^). Since (A = 1) cm^2^ in the present configuration, the numerical values of (*I*) and (*j*) are equivalent [33]. The gravimetric formic-acid production rate was calculated from the measured concentration, catholyte volume and electrolysis time Equation (5):

(5) $$\left[r_{\mathrm{HCOOH}}=\frac{C_{\mathrm{HCOOH}}V}{t}\right]$$

where (*r*_HCOOH_) is the formic acid production rate (mg h^−1^), (*C*_HCOOH_) is the formic-acid concentration (mg L^−1^), (*V*) is the catholyte volume (L), and (*t*) is the electrolysis time (h). For comparison with literature reports [34] normalized to electrode area, an area-normalized production rate may additionally be expressed as Equation (6):

(6) $$\left[r_{\mathrm{area}}=\frac{C_{\mathrm{HCOOH}}V}{\mathrm{tA}}\right]$$

with units of mg h^−1^ cm^−2^ or, after conversion, mmol h^−1^ cm^−2^.The molar formic acid production rate was calculated as Equation (7):

(7) $$\left[{\dot{n}}_{\mathrm{HCOOH}}=\frac{n_{\mathrm{HCOOH}}}{t}\right]$$

where $\left({\dot{n}}_{\mathrm{HCOOH}}\right)$ is the molar production rate (mol h^−1^), (*n*_HCOOH_) is the amount of formic acid produced (mol), and (*t*) is the electrolysis time (h). For convenient comparison with electrocatalytic literature, the value may also be reported in mmol h^−1^. The formic-acid partial current density was calculated from the total geometric current density and the FE as in Equation (8):

(8) $$\left[j_{\mathrm{HCOOH}}=j\left(\frac{FE_{\mathrm{HCOOH}}}{100}\right)\right]$$

where (*j*_HCOOH_) is the partial current density toward formic acid (mA cm^−2^), (*j*) is the total geometric current density (mA cm^−2^), and (*FE*_HCOOH_) is the FE for formic acid (%) [33]. To account for differences in catalyst loading, the catalyst-normalized productivity was calculated in Equation (9) by dividing the molar production rate by the mass of catalyst deposited on the electrode:

(9) $$\left[P_{\mathrm{cat}}=\frac{{\dot{n}}_{\mathrm{HCOOH}}}{m_{\mathrm{cat}}}\right]$$

where (*P*_cat_) is the catalyst-normalized productivity (mmol g^−1^ h^−1^); $\left({\dot{n}}_{\mathrm{HCOOH}}\right)$ is the molar formic acid production rate (mmol h^−1^), and (*m*_cat_) is the catalyst mass (g) [31,32].

The electrical energy requirement and energy efficiency of formic acid production were also evaluated to quantify the energetic performance of the electrochemical CO_2_ conversion process. The specific energy consumption (SEC) was calculated from the electrical energy supplied during the electrolysis and the corresponding mass of formic acid produced, as per Equation (10):

(10) $$\mathrm{SEC}=\frac{E_{\mathrm{elec}}}{m_{\mathrm{HCOOH}}}$$

where *SEC* is the specific energy consumption (kWh kg^−1^ HCOOH), *E*_elec_ is the electrical energy consumed (kWh), and *m*_HCOOH_ is the mass of formic acid produced (kg). The energy efficiency was estimated from the ratio of the chemical energy stored in the produced formic acid to the electrical energy supplied, as in Equation (11):

(11) $$\eta_{E}=\frac{m_{\mathrm{HCOOH}}\times \mathrm{HH}V_{\mathrm{HCOOH}}}{E_{\mathrm{elec}}}\times 100$$

where *η_E_* is the energy efficiency (%), *m*_HCOOH_ is the mass of formic acid produced (kg), *HHV*_HCOOH_ is the higher heating value of formic acid (5.52 MJ kg^−1^), and *E*_elec_ is the electrical energy input expressed in the same energy units as the numerator.

## 4. Conclusions

The Bi–O–S/acid-treated Sn electrode demonstrated enhanced electrochemical CO_2_ conversion to formic acid through the combined effects of Bi–O–S catalyst incorporation and acid-induced modification of the Sn substrate. Formic acid concentration increased from 170 to 750 mg L^−1^ with progressive substrate treatment and catalyst incorporation. Multi-objective optimization identified operating conditions that provided 1020 mg L^−1^ formic acid at 67% Faradaic efficiency (FE), with a corresponding production rate of approximately 41 mg h^−1^. The electrode maintained relatively consistent performance during the first 33 h of cumulative operation, although a decline in productivity and FE occurred during the subsequent cycle.

Further development should focus on improving FE, suppressing the competing hydrogen evolution reaction, and enhancing catalyst-layer adhesion and long-term stability. Optimization of catalyst loading, current density, and catholyte concentration, together with improved CO_2_ mass transfer, may further enhance productivity and selectivity. Future studies should combine extended electrolysis with pre- and post-operation surface and structural characterization to clarify the degradation mechanisms and guide the development of more durable electrodes. For practical or industrial implementation, larger electrode areas, continuous-flow operation, energy consumption, fabrication scalability, and performance reproducibility should also be evaluated under realistic operating conditions. Although these results demonstrate the potential of the Bi–O–S/acid-treated Sn electrode for CO_2_ electroreduction, further improvements in efficiency, durability, and scalability are necessary before practical implementation can be considered.

## Figures and Tables

**Table 1:** BET surface area and total pore volume of the Bi–O–S catalyst.

Parameter	Value
Bi content	62.42 wt%
O content	27.74 wt%
S content	9.84 wt%
BET surface area	3.921 m^2^ g^−1^
Total pore volume	0.001476 cm^3^ g^−1^

**Table 2:** Comparison of formic acid production using different Sn-based electrode configurations under identical electrochemical conditions.

Working Electrode	Catholyte Concentration (M)	Applied Current (mA)	Formic Acid Concentration (mg L^−1^)
Untreated Sn	0.60	−50	170
Acid-treated Sn (AtSn)			310
Bi–O–S/Sn			580
Bi–O–S/AtSn			750

**Table 3:** Central composite design matrix and experimentally measured formic acid concentration, FE, specific energy consumption, and energy efficiency.

Run	Applied Current (mA)	Catholyte Concentration (M)	Catalyst Dosage (mg)	Formic Acid Concentration (mg L^−1^)	FE (%)	Specific Energy Consumption (kWh kg^−1^ HCOOH)	Energy Efficiency (%)
1	45	0.45	2.0	623	65	3.61	42.46
2	20	0.20	3.0	260	61	3.85	39.87
3	70	0.20	3.0	525	35	6.67	23.00
4	70	0.70	3.0	411	27	8.52	18.01
5	20	0.70	3.0	300	70	3.33	46.00
6	45	0.45	4.5	462	48	4.87	31.48
7	45	0.03	4.5	493	51	4.56	33.60
8	45	0.45	4.5	698	73	3.22	47.57
9	3	0.45	4.5	49	76	3.06	50.09
10	45	0.45	4.5	746	78	3.02	50.84
11	45	0.87	4.5	557	58	4.04	37.96
12	45	0.45	4.5	656	68	3.43	44.71
13	45	0.45	4.5	538	56	4.18	36.66
14	45	0.45	4.5	700	78	3.21	47.70
15	87	0.45	4.5	863	54	5.04	30.42
16	20	0.20	6.0	312	73	3.21	47.84
17	20	0.70	6.0	317	74	3.15	48.61
18	70	0.20	6.0	948	63	3.69	41.53
19	70	0.70	6.0	842	56	4.16	36.89
20	45	0.45	7.0	448	47	5.02	30.53

**Table 4:** RSM-based optimization objectives predicted operating conditions, and experimental validation.

Optimization Case	Optimal Conditions (mA/M/mg)	Predicted FA (mg L^−1^)	Predicted FE (%)	Experimental FA (mg L^−1^)	Experimental FE (%)
Maximum FA concentration	87/0.26/7.0	1082	—	—	—
Maximum FE	3.0/0.63/3.4	—	85	—	—
Compromise optimum	73/0.33/6.5	950	61	1020	67

**Table 5:** Long-term electrochemical stability of the Bi–O–S/acid-treated Sn electrode.

Cycle	Duration (h)	Formic Acid (mg L^−1^)	FA Production Rate (mg h^−1^)	FE (%)
1 *	11	12,800	46.5	74
2 *	11	12,700	46.2	74
3 *	11	12,650	46.0	73
4 *	11	8500	30.9	49

* Fresh catholyte was used for each successive experiment with the same electrode.

**Table 6:** Comparison of representative Bi-based electrocatalysts for electrochemical CO_2_-to-formate/formic acid conversion.

Catalyst	Electrode/Cell	Catholyte (NaHCO_3_)	Potential (V vs. RHE)	Current Density (mA cm^−2^)	FE (%)	Productivity	Stability (h)	Ref.
Ultrathin Bi nanosheets	Bi nanosheets/H-cell	0.5 M	−1.50	16	95	NR	10	[53]
Bi–PMo nanosheets	Bi–PMo/electrochemical cell	0.5 M	−0.86	30	93	NR	NR	[54]
Cu foam@Bi nanowires	Cu foam@BiNW/H-cell	0.5 M	−0.69	15	95	NR	12	[55]
Bi_2_O_2_CO_3_-based Bi	Bi_2_O_2_CO_3_/H-cell	0.5 M	−0.90	11	95	NR	NR	[56]
Bi_2_O_2_CO_3_ nanosheets	Bi_2_O_2_CO_3_/H-cell	0.1 M	−0.70	9	85	NR	NR	[57]
Bi_2_S_3_-derived defect-rich Bi	Bi_2_S_3_-derived Bi/H-cell	0.5 M	−0.75	5	84	NR	NR	[58]
Activated Bi_2_Te_3_ nanoparticles/C	Bi_2_Te_3_-derived Bi/H-cell	0.5 M	−0.90	14	89	NR	NR	[6]
Bilayer Bi_12_O_17_Cl_2_ nanosheets	Bi-based electrode /H-cell	0.5 M	−0.89	11	93	NR	NR	[30]
**Bi–O–S/acid-treated Sn**	**Bi–O–S/AtSn/H-cell**	**0.33 M**	**Optimized condition**	**73**	**67**	**1020 mg L^−1^; 40.8 mg h^−1^**	**33** *	**This work**

NR = not reported. * 33 h represents the period over which relatively stable performance was retained during the first three consecutive 11 h cycles, with fresh catholyte used between experiments.

## Data Availability

The data used to support the findings of this study are available from the corresponding author upon request.

## Supplementary Materials

The following supporting information can be downloaded at: https://www.mdpi.com/article/10.3390/molecules31203718/s1. Supplementary Table S1: Predicted and experimentally measured values of formic acid concentration and FE obtained from the RSM model. Supplementary Figure S1: Parity plot showing experimental versus RSM-predicted formic acid concentration. Supplementary Figure S2: Parity plot showing experimental versus RSM-predicted FE. Supplementary Figure S3: Minitab plot showing the simultaneous optimization of formic acid concentration and FE.
