# Supplementary material for: Bi–O–S Catalyst for Enhanced CO2 Electroreduction to Formic Acid: Catalyst–Substrate Engineering and Multi-Objective Optimization
Source: Molecules. 2026 Oct 16;31(20):3718. doi: 10.3390/molecules31203718 (PMC13707935; doi:10.3390/molecules31203718)
Supplement: Supplementary file 1 [file molecules-31-03718-s001.zip › molecules-4616564-supplementary.pdf]

Supplementary files

Supplementary Table S1: Predicted and experimentally measured values of formic acid concentration and FE obtained from the RSM model.

Applied current (mA)	Catholyte concentration (M)	Catalyst dosage (mg)	Predicted FA Conc. (mg/L)	Experiment FA Conc. (mg/L)	Predicted FE (%)	Experiment FE (%)
45	0.45	2.0	457	623	50	65
20	0.20	3.0	301	260	65	61
70	0.20	3.0	597	525	41	35
70	0.70	3.0	527	411	38	27
20	0.70	3.0	364	300	74	70
45	0.45	4.5	644	462	67	48
45	0.03	4.5	531	493	53	51
45	0.45	4.5	644	698	67	73
3	0.45	4.5	96	49	81	76
45	0.45	4.5	644	746	67	78
45	0.87	4.5	514	557	55	58
45	0.45	4.5	644	656	67	68
45	0.45	4.5	644	538	67	56
45	0.45	4.5	644	750	67	78
87	0.45	4.5	811	863	49	54
20	0.20	6.0	205	312	63	73
20	0.70	6.0	254	317	68	74
70	0.20	6.0	894	948	60	63
70	0.70	6.0	811	842	53	56
45	0.45	7.0	613	448	61	47

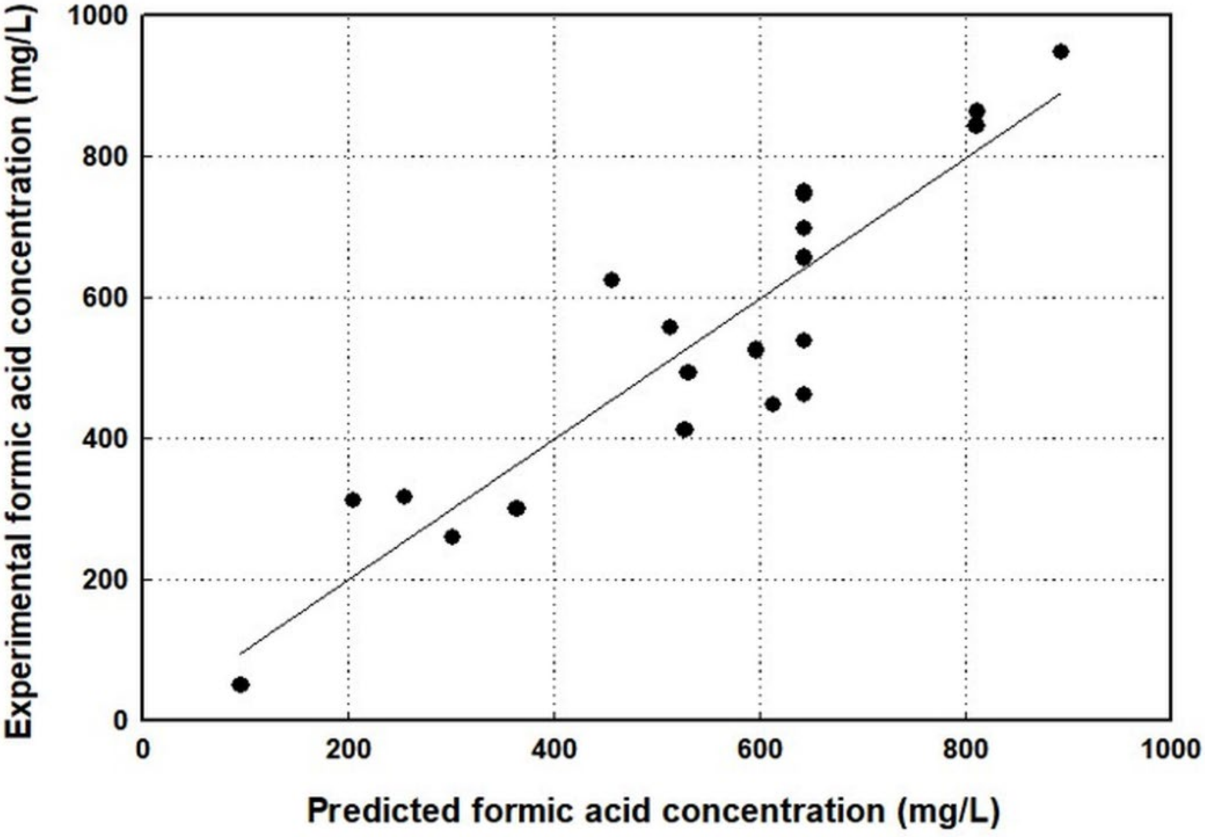

Supplementary Figure S1: Parity plot showing experimental versus RSM-predicted formic acid concentration

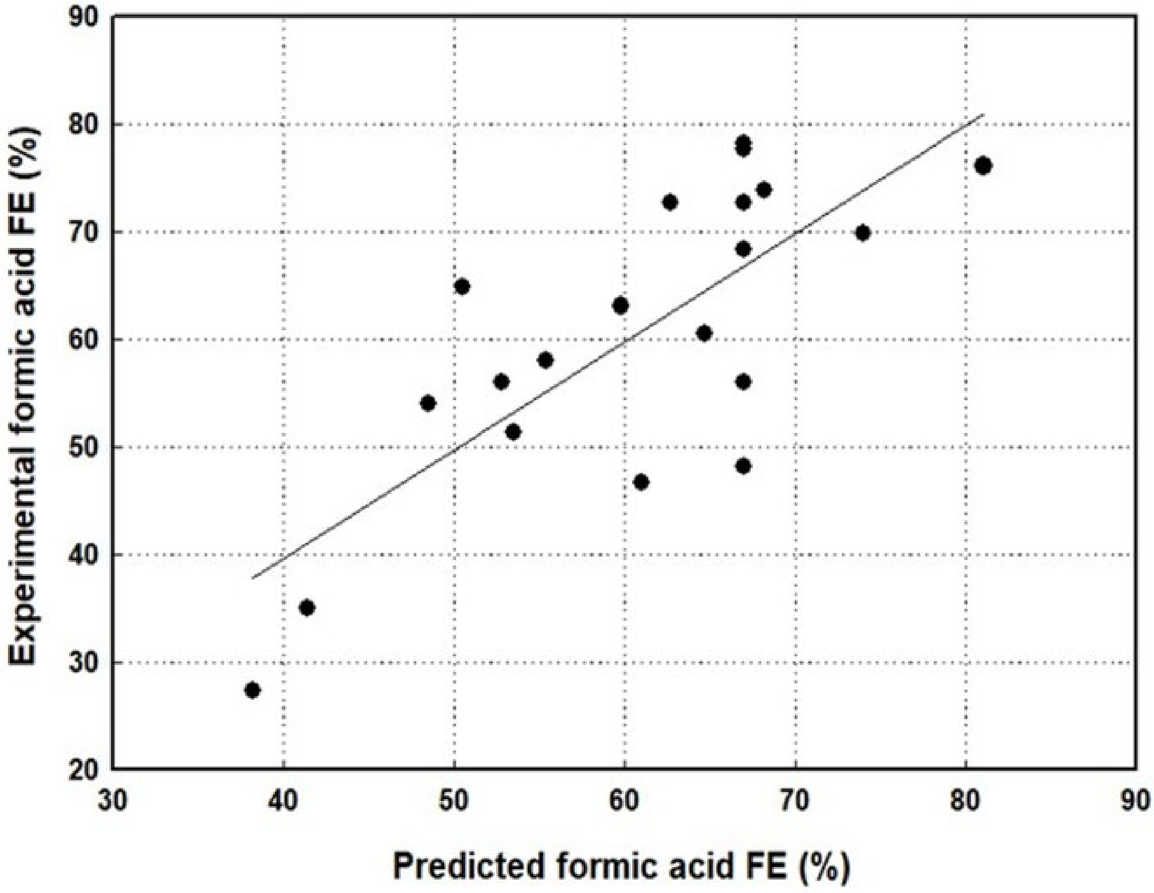

Supplementary Figure S2: Parity plot showing experimental versus RSM-predicted FE

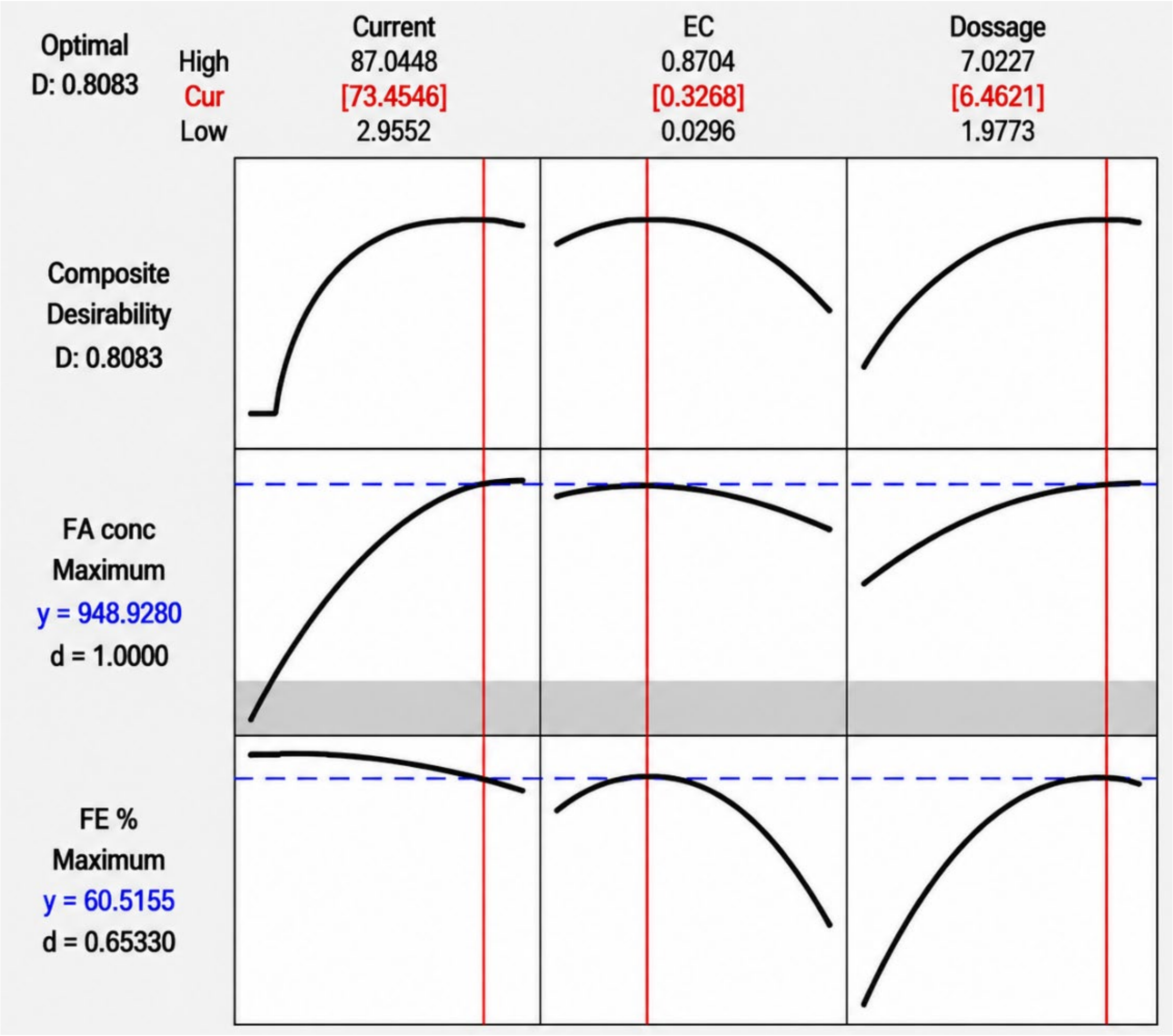

Supplementary Figure S3: Minitab plot showing the simultaneous optimization of formic acid concentration and FE.
